# Supplementary material for: Polarized anionic phospholipids and exocytosis are implicated in the polarized recruitment of budding yeast AP180, an endocytic initiator
Source: Mol Biol Cell. 2026 Apr 8;37(6):br17. doi: 10.1091/mbc.E24-10-0446 (PMC13200705; doi:10.1091/mbc.E24-10-0446)
Supplement: Supplementary file 1 [file mbc-37-br17-s001.pdf]

Supplemental Materials

Molecular Biology of the Cell

Marchando *et al*.

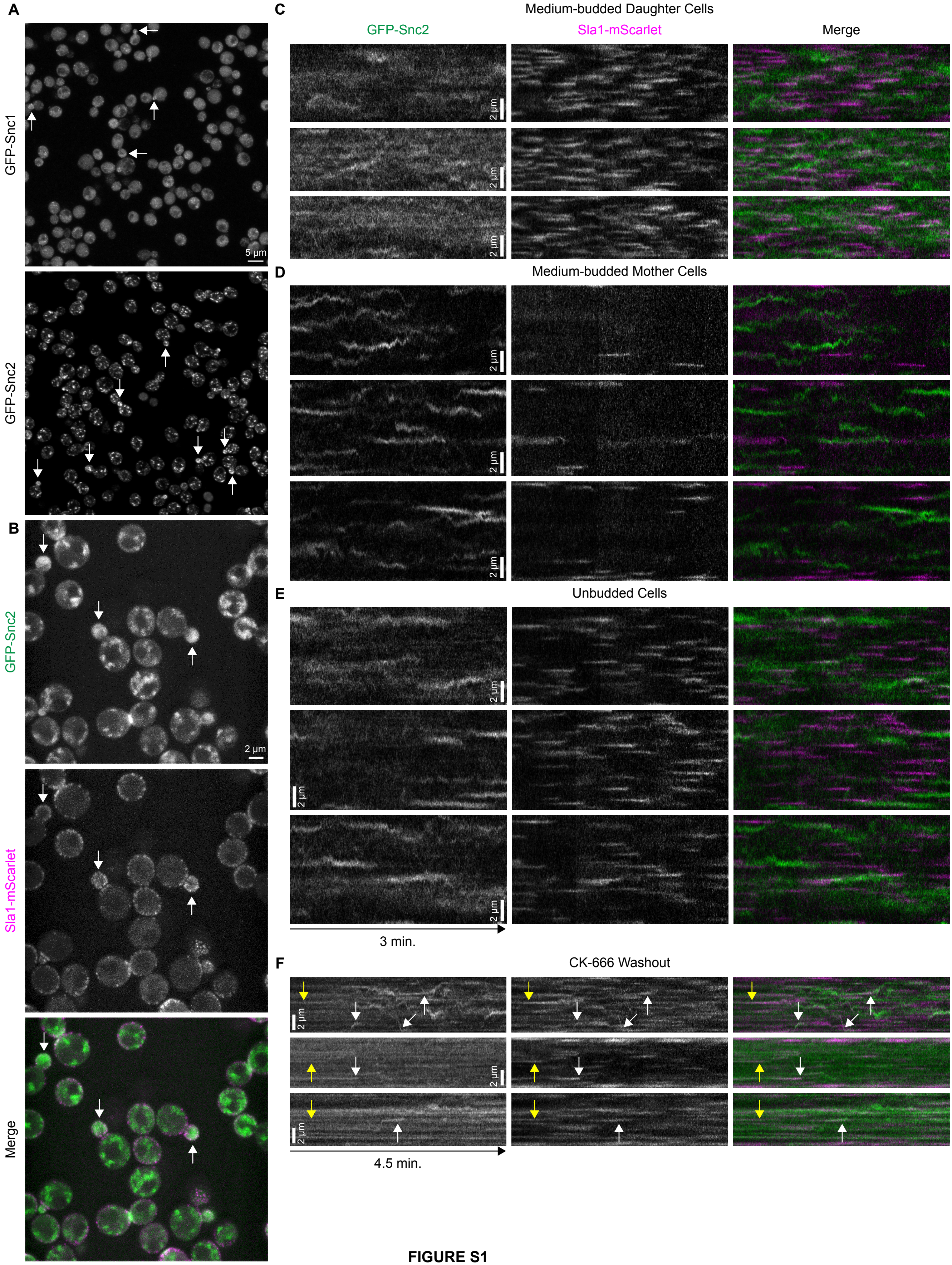
A
GFP-Snc1
5 µm
GFP-Snc2
B
GFP-Snc2
2 µm
Sla1-mScarlet
Merge
C
Medium-budded Daughter Cells
GFP-Snc2
Sla1-mScarlet
Merge
2 µm
D
Medium-budded Mother Cells
E
Unbudded Cells
3 min.
F
CK-666 Washout
4.5 min.

FIGURE S1

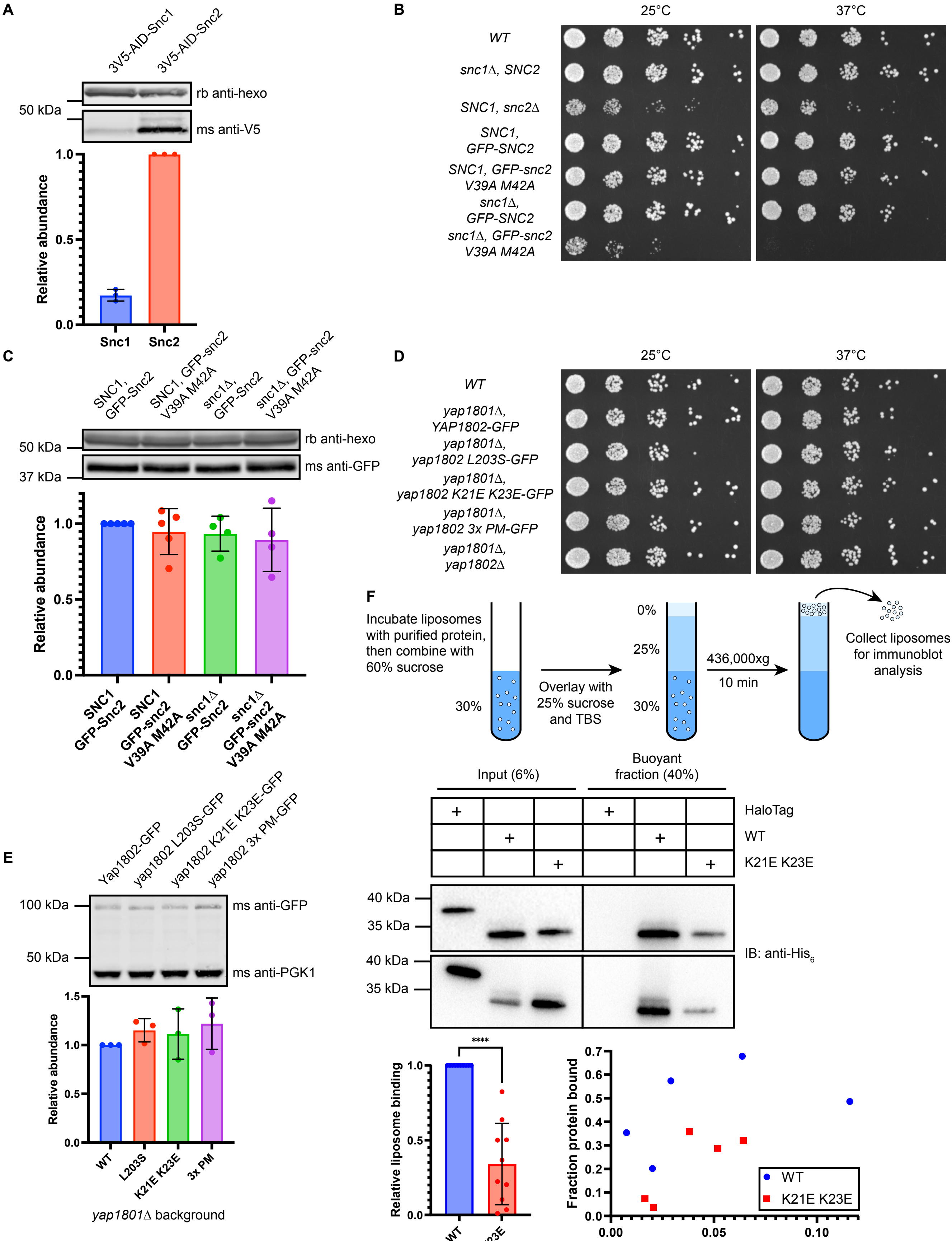
A
3V5-AID-Snc1
3V5-AID-Snc2
rb anti-hexo
50 kDa
ms anti-V5
Relative abundance
1.0
0.5
0.0
Snc1
Snc2
B
25°C
37°C
WT
snc1Δ, SNC2
SNC1, snc2Δ
SNC1, GFP-SNC2
SNC1, GFP-snc2 V39A M42A
snc1Δ, GFP-SNC2
snc1Δ, GFP-snc2 V39A M42A
C
SNC1, GFP-Snc2
SNC1, GFP-snc2 V39A M42A
snc1Δ, GFP-Snc2
snc1Δ, GFP-snc2 V39A M42A
50 kDa
rb anti-hexo
37 kDa
ms anti-GFP
Relative abundance
SNC1 GFP-Snc2
SNC1 GFP-snc2 V39A M42A
snc1Δ GFP-Snc2
snc1Δ GFP-snc2 V39A M42A
D
25°C
37°C
WT
yap1801Δ, YAP1802-GFP
yap1801Δ, yap1802 L203S-GFP
yap1801Δ, yap1802 K21E K23E-GFP
yap1801Δ, yap1802 3x PM-GFP
yap1801Δ, yap1802Δ
E
Yap1802-GFP
yap1802 L203S-GFP
yap1802 K21E K23E-GFP
yap1802 3x PM-GFP
100 kDa
ms anti-GFP
50 kDa
ms anti-PGK1
Relative abundance
1.5
1.0
0.5
0.0
WT
L203S
K21E K23E
3x PM
yap1801Δ background
F
Incubate liposomes with purified protein, then combine with 60% sucrose
30%
Overlay with 25% sucrose and TBS
0%
25%
30%
436,000xg
10 min
Collect liposomes for immunoblot analysis
Input (6%)
Buoyant fraction (40%)
HaloTag
WT
K21E K23E
40 kDa
35 kDa
IB: anti-His6
****
Relative liposome binding
WT
K21E K23E
Fraction protein bound
Concentration (µM)
0.00
0.05
0.10
WT
K21E K23E

FIGURE S2

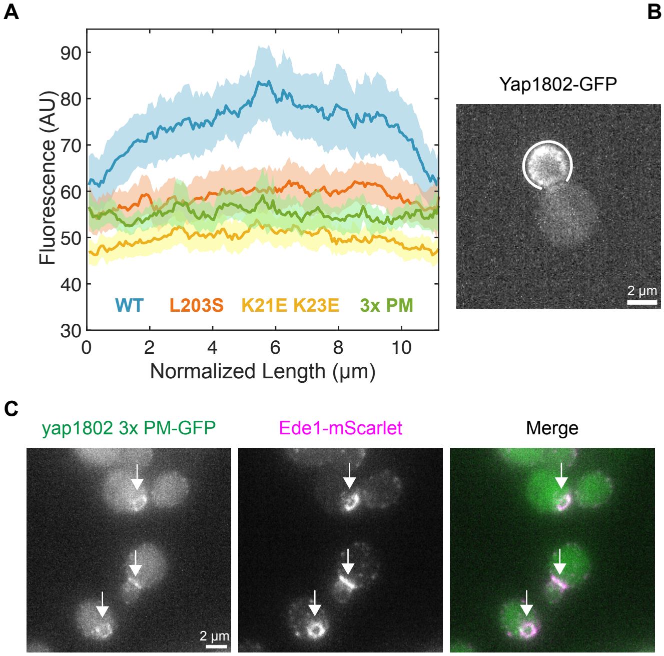
A
Fluorescence (AU)
Normalized Length (μm)
WT
L203S
K21E K23E
3x PM
Yap1802-GFP
2 μm
B
Fluorescence (AU)
Normalized Length (μm)
WT
L203S
K21E K23E
3x PM
mScarlet-Snc2
2 μm
C
yap1802 3x PM-GFP
Ede1-mScarlet
Merge
2 μm

FIGURE S3

FIGURE S1: A. Maximum intensity projections of Airyscan confocal z-stacks of fixed yeast cells expressing GFP-Snc1 (with intron removed from the *SNC1* gene) and GFP-Snc2, respectively. Budded cells are demarcated with arrows. **B.** Maximum intensity projections from a time-lapse NSPARC movie of cells expressing GFP-Snc2 and Sla1-mScarlet. Budded cells are demarcated with arrows. **C., D., E.** Three examples each of circumferential kymographs made from 3-minute NSPARC movies of cells expressing GFP-Snc2 and Sla1-mScarlet in daughter, mother, and unbudded cells, respectively. **F.** Circumferential kymographs made from a 4.5-minute NSPARC movie of daughter cells expressing GFP-Snc2 and Sla1-mScarlet acquired directly after washout of 50 µM CK-666 following 30 min. of treatment. White and yellow arrows denote distinct Snc2/Sla1 interaction events.

FIGURE S2: A. Immunoblot displaying relative levels of V5-AID-tagged Snc1 and Snc2 expressed from their normal promoters at their endogenous loci with anti-hexokinase loading control. **B.** Serial dilutions of strains carrying indicated *snc* null and point mutants and fluorescent fusion proteins on YPD. **C.** Immunoblot displaying relative levels of GFP-tagged Snc2 and snc2 V39A M42A mutant in *SNC1* and *snc1Δ* backgrounds. **D.** Serial dilutions of strains carrying various Yap1802 point mutations in a *yap1801Δ* background on YPD. **E.** Immunoblot displaying relative levels of GFP-tagged Yap1802 point mutants. **F.** Liposome flotation assays with purified truncated Yap1802 and yap1802 K21E K23E ANTH domains, two different replicates with different protein amounts loaded. Liposomes were made with a molar composition of 38% DOPC, 20% POPE, 20% DOPS, 3% PI(4,5)P_2, 0.5% TexasRed-PE, and 18.5% cholesterol. Relative binding was computed for each replicate with one exposure optimized for the input and one optimized for the buoyant fraction. Effective concentrations and binding fractions of the two ANTH proteins were determined using densitometry in relation to the known His_6-HaloTag concentration in each replicate using a single optimized exposure. Error bars for all immunoblots represent standard deviation. **** indicates $p < 0.0001$.

FIGURE S3: A. GFP line traces of daughter cells as in **C** from wild-type and Yap1802 point mutant maximum intensity projections for the same strains used in **Figure 2C**. **B.** mScarlet-Snc2 line traces from maximum intensity projections of mother cells as in **Figure 2G** expressing Yap1802 wild-type protein or point mutants. For each plot, each trace from individual cells was interpolated to match the mean length of cells in the wild-type cohort. Outlines represent a 95% confidence interval for each point in the trace, annotated microscopy images display examples of individual line traces used. **C.** Maximum intensity projections of widefield z-stacks of live cells expressing yap1802 3x PM-GFP and Ede1-mScarlet in a *yap1801Δ* background. Colocalization is denoted with arrows.

Supplemental Table 1

DDY Number	Alias	Mat	Base Genotype	Additional Genotype	Background
5943	PMY314.1	A	his3Δ200, ura3-52, leu2-3, 112, lys2-801(am)?	*GFP-SNC1 (no intron)::cgLEU2*	DDY1102
5944	PMY288.1	A	his3Δ200, ura3-52, leu2-3, 112, lys2-801(am)?	*GFP-SNC2::cgLEU2*	DDY1102
5945	PMY317.1	A	his3Δ200, ura3-52, leu2-3, 112, lys2-801(am)?	*3V5-AID2-SNC1 (no intron)::KanMX, TIR1::LEU2*	DDY1102
5946	PMY292.2	A	his3Δ200, ura3-52, leu2-3, 112, lys2-801(am)?	*3V5-AID2-SNC2::KanMX, TIR1::LEU2*	DDY1102
5947	PMY277.1	A	his3Δ200, ura3-52, leu2-3, 112, lys2-801(am)?	*snc1Δ::cgHIS3*	DDY1102
5948	PMY281.1	A	his3Δ200, ura3-52, leu2-3, 112, lys2-801(am)?	*snc2Δ::cgHIS3*	DDY1102
5949	PMY311.1	A	his3Δ200, ura3-52, leu2-3, 112, lys2-801(am)?	*GFP-SNC2::cgLEU2, snc1Δ::cgHIS3, SLA1-yomScarlet-I::KanMX*	DDY1102
5950	PMY312.1	α	his3Δ200, ura3-52, leu2-3, 112, lys2-801(am)?	*GFP-SNC2::cgLEU2, snc1Δ::cgHIS3, SLA1-yomScarlet-I::KanMX*	DDY1102
5951	PMY290.1	A	his3Δ200, ura3-52, leu2-3, 112, lys2-801(am)?	*GFP-SNC2 V39A M42A::cgLEU2*	DDY1102
5952	PMY409.1	A	his3Δ200, ura3-52, leu2-3, 112, lys2-801(am)?	*GFP-SNC2 V39A M42A::cgLEU2, YAP1802-yomScarlet-I::KanMX, snc1Δ::cgHIS3*	DDY1102
5953	PMY374.1	α	his3Δ200, ura3-52, leu2-3, 112, lys2-801(am)?	*YAP1802 L203S-GFP::cgLEU2, SLA1-yomScarlet-I::KanMX, yap1801Δ::NatR*	DDY1102
5954	PMY375.1	A	his3Δ200, ura3-52, leu2-3, 112, lys2-801(am)?	*YAP1802 L203S-GFP::cgLEU2, SLA1-yomScarlet-I::KanMX, yap1801Δ::NatR*	DDY1102
5955	PMY376.1	α	his3Δ200, ura3-52, leu2-3, 112, lys2-801(am)?	*YAP1802 K21E K23E-GFP::HIS3, SLA1-yomScarlet-I::KanMX, yap1801Δ::NatR*	DDY1102
5956	PMY377.1	A	his3Δ200, ura3-52, leu2-3, 112, lys2-801(am)?	*YAP1802 K21E K23E-GFP::HIS3, SLA1-yomScarlet-I::KanMX, yap1801Δ::NatR*	DDY1102
5957	PMY378.1	α	his3Δ200, ura3-52, leu2-3, 112, lys2-801(am)?	*YAP1802 K21E K23E L203S-GFP::cgLEU2, SLA1-yomScarlet-I::KanMX, yap1801Δ::NatR*	DDY1102
5958	PMY379.1	A	his3Δ200, ura3-52, leu2-3, 112, lys2-801(am)?	*YAP1802 K21E K23E L203S-GFP::cgLEU2, SLA1-yomScarlet-I::KanMX, yap1801Δ::NatR*	DDY1102
5959	PMY382.1	α	his3Δ200, ura3-52, leu2-3, 112, lys2-801(am)?	*YAP1802-GFP::HIS3, SLA1-yomScarlet-I::KanMX, yap1801Δ::NatR*	DDY1102
5960	PMY383.1	A	his3Δ200, ura3-52, leu2-3, 112, lys2-801(am)?	*YAP1802-GFP::HIS3, SLA1-yomScarlet-I::KanMX, yap1801Δ::NatR*	DDY1102
5961	PMY423.1	A	his3Δ200, ura3-52, leu2-3, 112, lys2-801(am)?	*YAP1802 K21E K23E L203S-GFP::cgLEU2, yomScarlet-I-SNC2::cgURA3, yap1801Δ::NatR*	DDY1102
5962	PMY441.1	A	his3Δ200, ura3-52, leu2-3, 112, lys2-801(am)?	*YAP1802-GFP::HIS3, yomScarlet-I-SNC2::cgURA3, yap1801Δ::NatR*	DDY1102
5963	PMY451.1	A	his3Δ200, ura3-52, leu2-3, 112, lys2-801(am)?	*YAP1802 L203S-GFP::cgLEU2, yomScarlet-I-SNC2::cgURA3, yap1801Δ::NatR*	DDY1102
5964	PMY452.1	α	his3Δ200, ura3-52, leu2-3, 112, lys2-801(am)?	*YAP1802 K21E K23E-GFP::HIS3, yomScarlet-I-SNC2::cgURA3, yap1801Δ::NatR*	DDY1102
5971	PMY462.1	α	his3Δ200, ura3-52, leu2-3, 112, lys2-801(am)?	*YAP1802 K21E K23E L203S-GFP::cgLEU2, EDE1-yomScarlet-I::KanMX, yap1801Δ::NatR*	DDY1102
5972	PMY463.1	A	his3Δ200, ura3-52, leu2-3, 112, lys2-801(am)?	*YAP1802 K21E K23E L203S-GFP::cgLEU2, EDE1-yomScarlet-I::KanMX, yap1801Δ::NatR*	DDY1102

Supplemental Table 2

pDD number	Name	Backbone	Resistance	Contents	Purpose	Competent Cells
pDD2745	pPM129	pFA6a	Amp	GFP-SNC2::cgLEU2	Longtine-style tagging plasmid	DH5α
pDD2746	pPM131	pFA6a	Amp	GFP-SNC2 V39A M42A::cgLEU2	Longtine-style tagging plasmid	DH5α
pDD2747	pPM133	pFA6a	Amp	3V5-AID2-SNC2::KanMX	Longtine-style tagging plasmid	DH5α
pDD2748	pPM134	pFA6a	Amp	GFP-SNC1 (no intron)::cgLEU2	Longtine-style tagging plasmid	DH5α
pDD2749	pPM135	pFA6a	Amp	3V5-AID2-SNC1 (no intron)::KanMX	Longtine-style tagging plasmid	DH5α
pDD2750	pPM150	pFA6a	Amp	YAP1802 L203S-GFP::cgLEU2	Longtine-style tagging plasmid	DH5α
pDD2751	pPM152	pUB1306	Kan	CAS9-GTCTTCATTATATACTAAGT (YAP1802 gRNA)::URA3 CEN	Yeast expression plasmid for YAP1802 editing mediated by CAS9	DH5α
pDD2752	pPM163	pFA6a	Amp	yomScarlet-I-SNC2::cgURA3	Longtine-style tagging plasmid	DH5α
pDD2753	pPM164	MacroLab 1-B	Kan	YAP1802 ANTH-6His	Expression vector for purification	DH5α
pDD2754	pPM165	MacroLab 1-B	Kan	YAP1802 ANTH K21E K23E-6His	Expression vector for purification	DH5α
